# The diagnostic efficacy and inappropriate biopsy rate of ACR TI-RADS and ATA guidelines for thyroid nodules in children and adolescents

**DOI:** 10.3389/fendo.2023.1052945

**Published:** 2023-03-27

**Authors:** Guanghan Li, Bo Zhang, Jia Liu, Ying Xiong

**Affiliations:** ^1^ Ultrasound Medical Department, China Japan Friendship Hospital, Beijing, China; ^2^ Department of Ultrasound, Civil Aviation General Hospital, Beijing, China

**Keywords:** ultrasound, thyroid nodule, fine needle aspiration, American College of radiology thyroid imaging reporting and data system, American thyroid association guidelines

## Abstract

**Background:**

This study is aimed at evaluating the diagnostic efficacy and unnecessary fine-needle aspiration (FNA) rate of ultrasound-based risk stratification for thyroid nodules in the American College of Radiology (ACR) Thyroid Imaging Reporting and Data System (TI-RADS) and the American Thyroid Association (ATA) risk stratification systems.

**Methods:**

Children and adolescents with pathology confirmed thyroid nodules were retrospectively included in this study. A total of 217 thyroid nodules from multicenter of Union Medical College Hospital, China Japan Friendship Hospital and Civil Aviation Hospital were included, the diagnostic efficiency and unnecessary FNA rate were calculated according to ACR and ATA guidelines.

**Results:**

Among all thyroid nodules, 139 nodules were malignant, and 78 nodules were benign. Choosing ATA high suspicion and ACR TI-RADS TR5 as benign and malignant cut-off points, the area under the curve and sensitivity of ATA were higher than ACR (AUC: 0.887 vs 0.840, p=0.0037; sensitivity 81.3% vs 71.0%, P <0.049;specificity 96.2% vs 97.4%, p=1.000;specificity both 85.9%); choosing high/intermediate suspicion in ATA and ACR TR4/5 as benign and malignant cut-off points, the two guidelines demonstrated similar diagnostic efficacy (AUC:0.890 vs 0.897, p=0.6038, sensitivity 92.1% vs 93.5%, P =0.817;specificity both 85.9%, p=1.000). The inappropriate FNA rate of ACR guideline was relatively lower (ATA 42.9% vs ACR 27.2%, P <0.001). If ACR TI-RADS TR5 nodules less than 1.0cm were included in the FNA indication, the unnecessary biopsy rate would be further reduced to 17.9%.

**Conclusion:**

This study indicated that both ATA and ACR TI-RADS risk stratification systems could provide a feasible differential diagnosis of benign and malignant thyroid nodules, while the ACR risk stratification system demonstrates a lower rate of inappropriate FNA rate. In addition, it was necessary to further study the minimum FNA threshold of thyroid nodules in Children and adolescents in order to reduce the missed biopsy rate of malignant nodules.

## 1 Introduction

The incidence rate of thyroid cancer in children and adolescents is relatively low, at approximately 1.9% of all thyroid cancers (1). While thyroid cancer is still the most common endocrine malignant tumor in children and adolescents, accounting for about 11% of all cancers in children and adolescents (2), its incidence rate is increasing yearly (3, 4). The pathological composition, ultrasonic imaging manifestations and biological characteristics of thyroid cancer in children and adolescents are also different from those in adults. The probability of metastasis and recurrence is higher. The metastasis rate can be as high as 40% - 80%, and the recurrence rate is about 30% (5, 6). Therefore, early diagnosis and treatment of thyroid cancer in children and adolescents are vitally important. Ultrasound is the first-line medical imaging choice for the diagnosis of thyroid nodules. At present, there are many thyroid grading diagnostic systems. For most grading diagnostic systems the nodule’s differentiation between benign and malignant and whether the nodules need to undergo ultrasound-guided fine needle aspiration cytology (FNA) was determined according to the ultrasonic characteristics of thyroid nodules and the size of the nodules. However, the current guidelines, such as the 2015 American Thyroid Association (7) (ATA for short), and ACR Thyroid Imaging Reporting and Data System (ACR TI-RADS for short) proposed in 2017 (8), were based on the ultrasonic characteristics of the adult thyroid nodules. More research was needed to verify the application of the above guidelines in children and adolescents with thyroid nodules (9). Therefore, this study aimed to compare the diagnostic efficacy and value for guiding FNA of ATA guidelines and ACR TI-RADS guidelines in children and adolescents.

## 2 Materials and methods

### 2.1 Patients

This study was retrospective. Patients with thyroid nodules younger than 18 years old were included. 178 patients who underwent thyroid surgery in Beijing Union Medical College Hospital from January 2000 to October 2017 were selected. Excluding 15 patients with incomplete clinical data, 11 patients with unclear ultrasound images, and 8 patients with no correspondence between ultrasound and pathological results, a total of 34 patients were excluded. 144 patient with 176 nodules were finally included. And 59 patients that had pathology results in China Japan Friendship Hospital and Civil Aviation General Hospital from November 2015 to January 2022 were selected, 12 patients had unclear images and 6 patients had no precise pathological results, a total of 18 patients were excluded, 41 patients with 41 nodules were finally included. All of the above included nodules have a clear pathological diagnoses.

### 2.2 Thyroid US examination and retrospective evaluation

Instrument: ultrasound examinations were performed by GE Logiq 9, Philips iU22, Philips IPQ7 and other color Doppler ultrasound diagnostic instruments, and the equipped probe frequency was 5-12 MHz.

**Figure 1:**
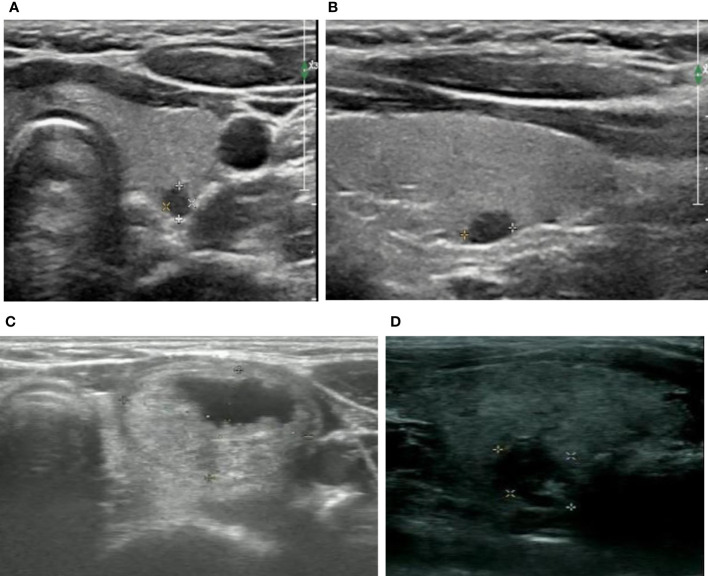
Ultrasound illustration of ATA and ACR TI-RADS pattern. **(A, B)** female, 18y, ACR TI-RADS 5 and ATA high suspicion, maximum diameter of 0.5cm, FNA and surgical pathology confirmed papillary carcinoma of thyroid **(C)** female, 16y, ACR TI-RADS 2 FNA not recommended;ATA low suspicion FNA recommended;maximum diameter of 2.2cm, surgical pathology confirmed nodular goiters **(D)** female,16y,ACR TI-RADS 4 FNA not recommended;ATA intermediate suspicion FNA recommended;maximum diameter of 1.2cm, FNA pathology confirmed inflammatory nodule.

Examination method and ultrasonic image evaluation method: The physician performed all examinations. During the examination, the patient took a supine position, fully exposed the neck, instructed the patient to breathe calmly, placed the probe gently in the thyroid region, and adjusted the gain, depth, image focus and other related parameters at any time to obtain the best imaging effect. The ultrasound physician routinely scanned the thyroid and bilateral cervical lymph nodes, and the ultrasonic examination data of all nodules were stored. Two radiologists specialized in ultrasound diagnosis with more than 5 years of experience retrospectively analyzed the ultrasound images of thyroid nodules, recorded the size, location, structure, echo, shape, edge, calcification, relationship with capsule of the nodules, and evaluated the nodules using the 2017 ACR TI-RADS (8) and the 2015 ATA (7) guideline classification diagnostic criteria. Both radiologists were blinded to final pathology and abnormal lymph nodes. If a patient had multiple nodules, each nodule should be analyzed separately. If they disagreed, the final judgment was discussed and negotiated. Ultrasound illustration of ATA and ACR TI-RADS pattern could be found in **Figure 1**.

### 2.3 Statistical analysis

The age and nodule size of patients was expressed by x ± S and interquartile ranges (IQR). The comparison of age between benign group and malignant group was analyzed by t test, and the comparison of sex adopted chi-square test. According to the recommendations of ACR TIRADS and ATA guidelines on biopsy, the nodules were divided into two groups, with pathological results as the “gold standard”, the receiver operator characteristic (ROC) curve was established. The sensitivity, specificity, positive predictive value, negative predictive value, accuracy and 95% confidence interval were calculated to evaluate the differential diagnostic effectiveness of the two guidelines. The inappropriate biopsy for thyroid nodules was defined as the number of benign nodules requiring biopsy and the number of malignant not requiring biopsy. SPSS 25.0 (IBM, Armonk, New York, USA) and MedCalc 11.4.2.0 software (MedCalc Software, Ostend, Belgium) were used for data analysis in this experiment, P<0.05 was statistically significant.

## 3 Results

### 3.1 Comparison of general data between benign group and malignant group

Among 217 thyroid nodules, 139 were malignant(including 131 papillary carcinoma, 7 follicular carcinoma and 1 undifferentiated carcinoma) and 78 were benign(including 53 nodular goiters, 5 inflammatory nodules, 10 adenomas, 2 hyperthyroidism changes after treatment, 1 fibrous hyperplasia and 7 FNA benign). Of all the nodules, 202 of them had surgical pathology and 15 of them had FNA pathology. The mean age of patients in malignant group was not significantly different from that in benign group [(15.47 ± 2.27) years vs (15.79 ± 2.47) years, P=0.401]. The proportion of male patients in malignant group and benign group was less than that of female patients, with a statistically significant difference [54 (87.1%) vs 86 (73.5%), P=0.038]. The size of nodules in malignant group was significantly smaller than that in benign group [(1.93 ± 1.11) cm vs (2.58 ± 1.20) cm, P<0.001] **Table 1**. The diagnosis performance of ATA and ACR TIRADS could be found in **Table 2**–**8**, performance as indication for FNA could be found in **Table 9**, **10**.

**Table 1:** Summary of demographic features.

	Pathological Findings		*P*
	Benign	Malignant	
Number of nodules	78	139	
Age(x ± s)	15.47 ± 2.27, IQR 14-18	15.79 ± 2.47, IQR 15-18	0.401
Sex			0.038
Male (%)	8(12.9%)	31 (26.5%)	
Female(%)	54(87.1%)	86 (73.5%)	
Nodule size(cm, x ± s)	2.58 ± 1.19, IQR 1.57-3.60	1.94 ± 1.11, IQR 1.10-2.70	<0.001

**Table 2:** Summary of ACR TIRADS and ATA grading table of nodules.

Pathology	ATA				ACR TIRADS			
	Very low suspicion	Low suspicion	Intermediate suspicion	High suspicion	2	3	4	5
Benigh	19	48	8	3	41	26	9	2
Malignant	0	11	15	113	0	9	32	98

The cancer rate of TR2 was 0%, TR3 was 25.71%, TR4 was78.05%, TR5 was 98%; The cancer rate of Very low suspicion was 0%, Low suspicion was 18.64%, Intermediate suspicion was 65.22%, High suspicion was 97.41%.

**Table 3:** Comparison of diagnostic efficacy between the diagnostic criteria of ACR TI-RADS TR5 and ATA high suspicion as malignant.

Pathological Findings	ATA		ACR TIRADS	
	Malignant	Benign	Malignant	Benign
Malignant	113	26	98	41
Benign	3	75	2	76

**Table 4:** Comparison of sensitivity, specificity, positive predictive value, negative predictive value, and accuracy of malignant tumors according to ATA guideline high suspicion and ACR TI-RADS TR5.

	Sensitivity	Specificity	Positive predictive value	Negative predictive value	Accuracy
ATA	113/139 (81.3%)	75/78 (96.2%)	113/116 (97.4%)	75/101 (74.3%)	188/217 (86.6%)
ACR TI-RADS	98/139 (71.0%)	76/78 (97.4%)	98/100 (98.0%)	76/117 (62.0%)	174/217 (80.2%)
P	0.049	1.000	1.000	0.144	0.093

The above results showed that ATA guideline is more sensitive than ACR guideline, but there is no significant statistical difference in specificity, positive predictive value, negative predictive value and accuracy.

**Table 5:** Comparison of area under curve of malignant tumors according to ATA guideline high suspicion and ACR TIRADS TR5.

	AUC	SE^a^	95%CL^b^	Z	p
ATA	0.887	0.0199	0.837-0.926	2.906	0.0037
ACR TI-RADS	0.840	0.0214	0.784-0.886		

The area under the curve of ATA guideline was higher than that of ACR TI-RADS (0.887 vs 0.840), P=0.0037. ^a^DeLong's test. ^b^Binomial precision.

**Table 6:** Comparison of diagnostic efficacy between the diagnostic criteria of ACR TI-RADS TR4/5 and ATA high/intermediate suspicion as malignant.

Pathological Findings	ATA		ACR TI-RADS	
	Malignant	Benign	Malignant	Benign
Malignant	128	11	130	9
Benign	11	67	11	67

**Table 7:** Comparison of sensitivity, specificity, positive predictive value, negative predictive value, and accuracy according to ACR TI-RADS TR4/5 and ATA high/intermediate suspicion as malignant.

	Sensitivity	Specificity	Positive predictive value	Negative predictive value	Accuracy
ATA	128/139 (92.1%)	67/78 (85.9%)	128/139 (92.1%)	67/78 (85.9%)	195/217 (89.9%)
ACR TI-RADS	130/139 (93.5%)	67/78 (85.9%)	130/141 (90.8%)	67/76 (92.2%)	197/217 (90.8%)
P	0.817	1.000	1.000	0.812	0.871

The above results show that there was no significant statistical difference in sensitivity, specificity, positive predictive value, negative predictive value and accuracy between ATA guidelines and ACR guidelines.

**Table 8:** Comparison of area under curve of malignant tumors according to ACR TI-RADS TR4/5 and ATA high/intermediate suspicion as malignant.

	AUC	SE^a^	95%CL^b^	Z	p
ATA	0.890	0.0229	0.841-0.928	0.519	0.6038
ACR TI-RADS	0.897	0.0224	0.849-0.934		

There was no significant statistical difference (P=0.6038) between ACR TIRADS TR4/5 and ATA high/intermediate suspicion as malignant (P=0.6038). While there was also no significant statistical difference between the high suspicion of ATA guidelines and intermediate suspicion of ATA guidelines and ACR TI-RADS guidelines TR4 and TR5 (P=0.9021 and P=0.6568).The above results showed that ATA high suspicion and intermediate suspicion, and ACT TIRADS TR4 and TR5 are malignant, with similar overall diagnostic efficacy. ^a^DeLong's test. ^b^Binomial precision.

**Table 9:** The application guidelines recommend threshold, ATA high suspicion/TR5 nodule nodule removal greater than 1cm threshold, and compare the value of the above guidelines in guiding FNA.

Pathological Findings	ATA		ACR TI-RADS		ATA high suspicion removal greater than 1cm threshold		ACR TI-RADS5 removal greater than 1cm threshold	
	FNA	No FNA	FNA	No FNA	FNA	No FNA	FNA	No FNA
Malignant	108	31	105	34	133	6	128	11
Benign	60	18	22	56	61	17	23	55

**Table 10:** Inappropriate FNA rate, missed FNA rate of malignant nodules, FNA rate of benign nodules and area under the curve of recommended threshold and optimized threshold in the application guideline.

	Combined inappropriate FNA and missed FNA	Missed FNA of malignant nodules	Inappropriate FNA of benign nodules	AUC
ATA	91/217 (41.9%)	31/139 (22.3%)	60/78 (76.9%)	0.504
ACR TIRADS	56/217 (25.8%)	34/139 (24.5%)	22/78 (28.2%)	0.737
ATA high suspicion removal greater than 1cm threshold	67/217 (30.9%)	6/139 (4.3%)	61/78 (78.2%)	0.587
ACR5 removal greater than 1cm threshold	34/217(15.7%)	11/139(7.9%)	23/78 (29.5%)	0.813

Inappropriate FNA was defined as the number of benign nodules punctured. A statistical difference existed between two AUCs of the above four standards (P<0.001). The accuracy of using the ACR TI-RADS guidelines to guide FNA was higher than that of ATA guidelines. After the ACR TI-RADS guidelines relaxed the limit of 1cm for TR5 nodules, the efficiency of guiding FNA was further improved, with the combined inappropriate FNA and missed FNA rate reduced to 15.7%, the malignant nodule missed FNA rate reduced to 7.9%, the inappropriate FNA rate of 29.5%, and the area under the curve increased to 0.813.

## 4 Discussion

Ultrasound is the first-line imaging method for thyroid diseases. The grading system can be used to evaluate the benign and malignant nodules by standard methods, reduce the difference between observers, and guide whether the nodules have indications of FNA (10). The most widely used ATA and ACR TI-RADS guidelines are based on ultrasonic image characteristics and nodule size to determine the risk stratification of nodules and whether FNA is recommended. However, these two the guidelines are based on adult ultrasound data. There were still controversy about the diagnostic efficacy of the above guidelines in adults’ and children’s thyroid nodules. There were also large differences in the sensitivity and specificity data obtained from different studies (11–13 ), the sensitivies of different reports were between 0.37-1.00, and the specificities were between 0.24-0.90 (9, 14, 15). Therefore, applying the diagnostic grading diagnostic system in children and adolescents’ thyroid nodules deserves further research.

Different studies chose different cutoff points for differentiating benign and malignant tumors. This study showed that the specificity was relatively higher when selecting high suspicion in the ATA and TR5 as malignant compared to those choosing high/intermediate suspicion in the ATA and TR4/5 in ACR TI-RADS, the specificity was relatively higher (96.2% and 97.4% vs. 85.9% and 85.9%). The sensitivity was relatively lower (92.1% and 93.5% vs. 81.3% and 71.0%). Nevertheless, there was no significant difference in the area under the curve between ATA high suspicion, ACR TR5 and ACR TR4/5(0.887 vs 0.890 vs 0.897). It showed that the above two guidelines had similar overall diagnostic efficacy, which was similar to some previous studies (13, 16, 17). Although these two guidelines had similar overall diagnostic efficacy, appropriate diagnostic thresholds could be selected in our daily work to obtain higher diagnostic sensitivity or specificity.

ATA guidelines and ACR TI-RADS guidelines would not propose to recommend FNA for most thyroid nodules<1cm (8, 9), but previous studies had shown that different nodule FNA thresholds had a great impact on the guidance of nodules (18–20). Different guidelines differed in terms of the minimum nodule FNA size. For example, the French thyroid guidelines set the minimum FNA size as 7mm (21). Currently, studies have shown a high rate of missed FNA of malignant nodules and inappropriate FNA of benign nodules in children and adolescents using ACR and ATA guidelines for thyroid nodules (22). As thyroid cancer in children and adolescents was more malignant than that in adults, with a higher probability of local invasion and metastasis, early diagnosis was crucial to managing of nodules (23). In the 2015 ATA Guidelines for Children’s Thyroid, it was pointed out that it is difficult to deal with thyroid nodules smaller than 1cm in children’s thyroid, because the volume of children’s thyroid increases with age, and the size of individual nodules had little significance in predicting benign and malignant tumors. Therefore, when determining FNA criteria, we should refer to the ultrasonic characteristics of nodules and whether there are high-risk factors in patients (24). In this study, thyroid nodules smaller than 1cm accounted for 21.2% (46/217) of all nodules, so the minimum FNA size of TR5 and ATA high suspicion nodules with the highest risk classification was reduced in this study because these nodules had the most apparent ultrasonic malignant characteristics. After reducing the minimum size of nodule FNA, the rate of missed FNA of malignant nodules was significantly reduced, and the area under the curve was further improved.

As for the limitations of this study, first of all this study was a retrospective study, which inevitably had a particular selection bias. Radiologists assessed nodules and reached consensus, which could lead to higher accuracy than “real world”. A larger sample of clinical studies could be conducted to provide more information for diagnosis and the revision of the guideline FNA threshold. Second, the proportion of benign nodules in the included subjects was relatively small, while the malignant rate was high. That was mainly because the inclusion criteria of this study was that nodules with precise pathological results, so the diameter of the included benign nodules was larger, and the average diameter of benign nodules was larger than that of malignant nodules. In addition, only the nodules with definite pathology were included in this stud. This study did not include many benign nodules requiring follow-up but no need for FNA. Third, although this study showed that the rate of inappropriate FNA and the rate of missed FNA of malignant nodules had been reduced after the high suspicion nodule less than 1cm included, further research was still needed on the minimum threshold, FNA accuracy of nodules less than 1cm and whether the prognosis of patients after FNA could be improved,.

## 5 Conclusion

In conclusion, ATA and ACR TI-RADS guidelines were similar in diagnosing benign and malignant nodules in children and adolescents. ACR TI-RADS guidelines had higher accuracy in guiding FNA. In addition, further research was needed to determine the FNA threshold of thyroid nodules in children and adolescents to improve the overall accuracy of FNA.

## Data availability statement

The raw data supporting the conclusions of this article will be made available by the authors, without undue reservation.

## Ethics statement

The studies involving human participants were reviewed and approved by China-Japan Friendship Hospital Ethics Committee. Written informed consent to participate in this study was provided by the participants’ legal guardian/next of kin. Written informed consent was obtained from the individual(s), and minor(s)’ legal guardian/next of kin, for the publication of any potentially identifiable images or data included in this article.

## Author contributions

GL and BZ participated in the study design, performed the statistical analysis and drafted the manuscript. JL and YX participated in its design and collection of samples. BZ participated in review of manuscript. All authors read and approved the final manuscript. All authors contributed to the article and approved the submitted version.
